# The costs of celiac disease: a contingent valuation in Switzerland

**DOI:** 10.1007/s10198-021-01376-z

**Published:** 2021-10-07

**Authors:** Laia Soler, Nicolas Borzykowski

**Affiliations:** 1 University of Lausanne, Lausanne, Switzerland; 2 University of Applied Sciences and Arts Western Switzerland in Business Administration (HEG-Genève), Geneva, Switzerland

**Keywords:** Celiac disease, Contingent valuation, Gluten-free diet, Intangible costs, Cost-of-illness, I11, I18

## Abstract

This paper proposes a first monetary measure of the private costs of celiac disease, including intangible costs (physical symptoms, logistical constraints, etc.) in Switzerland. This auto-immune disease damages the intestine when patients ingest gluten. The only treatment currently available is a gluten-free diet, which implies great nutritional constraints. To get a monetary equivalent of the costs borne by celiac patients, we used a contingent valuation. The scenario suggested to celiac patients a treatment in form of a daily pill, which would allow them to eat normally and avoid any physical pain from celiac disease. Mean Willingness To Pay (WTP) for the treatment is found to be around CHF 87 (approx. USD 87) per month. WTP is positively influenced by direct and indirect costs of the disease. Oppositely, individuals, who find the gluten-free diet healthier are willing to pay less. Finally, unlike symptoms before diagnostic, the current presence or intensity of physical symptoms are found to be insignificant. The latter result can be explained by the fact that, individuals facing stronger symptoms are more likely to adhere strictly to the GFD and hence to reduce their frequency.

## Introduction

Celiac disease is a chronic auto-immune disease that appears in genetically predisposed individuals. It causes an atrophy of the intestinal villi in reaction to the ingestion of gluten, contained in wheat, barley, rye, and their derivatives [17]. Celiac disease comes with a variety of intestinal and extra-intestinal symptoms. Its classical presentation generally appears at the infant’s age, when gluten is first introduced in the diet. Children will, for example, present malabsorption or a failure to thrive. In the adult population, the disease is more likely to show atypical symptoms. It is estimated that about 1% of the European population suffers from celiac disease, a prevalence that seems to be increasing in Western countries [34]. However, as the symptoms are varied, the disease is frequently underdiagnosed. Indeed, according to Catassi et al. [14], only 1 case out of 3–5 is diagnosed.

The only treatment is a strict gluten-free diet (GFD). In general, food with less than 20 particles per million (ppm) is considered harmless for celiac patients [7], which is equivalent to 20 mg per kilogram of food, roughly the tip of a pen. In theory, this treatment is quite straightforward but in practice, it is very hard to follow, as gluten is an important component of our western diet. Furthermore, wheat and other gluten-containing flours are very volatile and thus easily contaminate naturally gluten-free food. This problem of “cross-contamination” extends to the kitchen, where it becomes difficult to guarantee a gluten-free meal when other gluten-containing meals are prepared in the same room.

Living with celiac disease can be difficult as it might cause unpleasant symptoms and concerns. Celiac patients show a higher risk for certain infections such as tuberculosis, influenza or clostridium difficile (see [27]). In addition, the treatment consisting of a strict GFD can be burdensome. Different studies have illustrated the difficulties associated with the diet. A majority of patients mentions in a recent study from the U.S [51] that the GFD affects them socially and reports difficulties when eating out. For example, it might become more difficult to be invited for dinner, as friends might not know how to prepare a gluten-free meal or might not want to take the risk. Additionally, the fact that celiac patients often have their own special food can be felt as a kind of exclusion. Patients often mention feeling different, the fear of exclusion or loneliness in their struggle [21, 51, 54]. Therefore, both the disease and its treatment can be sources of psychological and social suffering [21, 33, 45, 51]. This suffering, the symptoms and the GFD impose direct, indirect and intangible costs on patients. These costs could be avoided if there was a simple pill to cure celiac disease.

The OECD [37] accounts for several types of costs related to a disease. These costs are supported either by the patient herself and/or by the society as a whole. In the case of celiac disease (Table 1), the private direct costs correspond to the medical expenses of the diagnosis and those that occur afterwards, as well as the supplementary cost attached to GFD. The public also supports part of the direct medical costs through the system of social insurance. Indirect costs comprise a loss of productivity in the presence of symptoms and are borne by both society and the individual. A loss of time due to additional time spent on cooking or grocery shopping can also occur. Intangible costs comprise physical pain, psychological costs and social costs. The latter type of costs is the most difficult to measure in monetary terms and is often ignored or unquantified [22].

**Table 1:** Classification of the costs for a cost-of-illness study

	Direct	Indirect	Intangible
Private	Medical expenses Increased food and beverages expenses	Loss of productivity Increased cooking time Increased logistical costs	Physical pain Psychological costs Social costs
Public	Transfer payments (insurances)	Loss of productivity Absenteeism Withdrawal from the work supply	

Some direct costs, such as the premium paid for gluten-free food, and indirect costs such as the extra time spent cooking and organizing and the loss of productivity have been specifically assessed for the case of Switzerland in Soler [48]. Some other costs, such as symptoms, were also described, but not monetarily quantified.

The present study estimates the total private costs related to celiac disease in Switzerland, thanks to a contingent valuation (CV). The CV method is a stated preferences method, which is widely used in environmental economics [19]. It allows valuing items, which do not have a market. Given the very nature of celiac disease’s intangible costs, this method is arguably a good tool to get a comprehensive estimation of the disease’s private costs. In this paper, we measure how much patients would be willing to pay for a hypothetical medicine that would treat celiac disease without the drawbacks of the current treatment (i.e., the GFD). Assuming patients truly reveal their maximum willingness to pay (WTP), they will be indifferent between foregoing this amount of money and keeping the current disease burden. Indeed, patients would be willing to purchase this treatment if the costs of the treatment does not exceed its opportunity costs: the costs of the disease. This equivalence can be used to represent the costs of the disease, including its intangible costs, in monetary terms [11].

Assessing the costs of a disease is crucial in a cost–benefit framework. First, a measure of costs can provide policy-makers with arguments to encourage a better labeling of food products and foster the provision of gluten-free food. The food industry could also consider these results to shape its gluten-free food pricing or extend its gluten-free products choice. Finally, a measure of WTP is an interesting tool for the pharmaceutical industry, which could decide to invest in research and development if WTP covers the costs. This measure could also help assessing the evolution of CD’s burden over time and evaluate whether given policy measures have an impact on patients’ well-being.

The remainder of the paper is structured as follows: Sect. Literature review presents the literature on the topic, Sect. Survey design introduces the methodology, Sect. Descriptive statistics describes the sample used, Sect. Empirical analysis presents the results, and Sect. Results discusses and concludes.

## Literature review

Other studies have used CV to estimate the burden of a disease or its treatment. For example, Lin et al. [30] assess the value of a treatment addressing multiple symptoms associated with multiple sclerosis. They use the results to understand which symptoms are more burdensome and guide treatment priorities. Using a dichotomous-choice format, they find that patients are, in general, willing to pay more than their neurologists think. Amounts range between 375 and 520 USD per month for treatments improving mobility, eyesight, cognition or upper limb function.

Li et al. [29] asked patients and their relatives how much they were willing to pay for a prostate cancer treatment without side effects, which is similar to our approach. The WTP was extracted with a table where respondents had to indicate how likely they were to pay the 9 different suggested amounts. Patients were willing to pay on average around USD 400 per month.

Beikert et al. [4] assessed patients’ WTP for a complete cure of atopic dermatitis and compare patients’ quality of life with other skin diseases. Similarly to celiac disease, this chronic disease also lacks a treatment. The authors use contingent valuation on patients throughout Germany and elicit the WTP in three different ways: with an open question for the absolute value of a complete cure, the monthly payment for a complete cure among 9 suggested amounts and the share of the monthly income while the payment lasts among 7 suggested answers. Results show that patients were willing to pay up to EUR 1000 (approx. USD 1200) for a complete cure, or 10–20% of their monthly income (all values correspond to the median). Interestingly, WTP is weakly correlated with factors affecting the quality of life. The only significant factor explaining WTP is the involvement of facial affections: patients that are more facially affected are willing to pay more than others. Other factors, which are relevant to predict quality of life such as skin dryness, sleep disturbances, pruritus, genital involvement or affected body surface, do not significantly explain WTP.

Studies suggest that different factors might be affecting the well-being of celiac patients and thus their WTP, namely adherence to the diet [26, 51], presence of symptoms [42], delay to diagnosis [16], time from diagnosis [44] support from close friends and family [21, 45, 51] and gender [52].

In several studies, however, celiac patients did not seem to have a lower health-related quality of life compared to the general population [52] [Canada]; [13] [Spain]; [18] [U.K.]; [34] [Sweden]; [43] [Finland]. But other studies show significant burden weighs on celiac patients [21, 33, 45, 51]. In general, studies based on generic indicators of health-related quality of life tend to show little burden from CD, whereas indicators specific to the disease uncover a significant burden. This may be due to the specificity of CD, which, when the treatment is followed, affects social relations or logistics rather than the physical well-being of the patients. Contingent valuation is a generic indicator since it values the burden in terms of a monetary amount. However, the respondent does not have to attribute the amount to certain specific aspects of her well-being. It could, therefore, allow a comparison with other diseases on a much broader base.

WTP could also be positively affected by the magnitude of direct and indirect costs foregone by patients and their income. For example, a person spending a high amount on gluten-free food might be more willing to pay for an alternative treatment. The same applies to someone who considers they would have better job opportunities without living with negative consequences of the disease.

Based on the literature, which measures quality of life for celiac patients mentioned above, we expect patients experiencing strong symptoms to be willing to pay more and those with supportive friends and relatives to be willing to pay less. The effect on WTP of the extent to which patients adhere to the diet is ambiguous. Patients could be adhesive because they appreciate the GFD—in which case this should decrease WTP—or because they are subject to strong symptoms following the absorption of gluten—in which case this should increase WTP.

To our knowledge, only Norström et al. [36] have used CV on celiac disease. They asked Swedish parents their WTP to test their child for celiac disease (screening strategy) in an open-ended format. The mean WTP was higher than the unit cost per child, suggesting that a screening strategy would be cost effective. However, the median was much lower than the mean and the unit cost. This questions the acceptability of a publicly financed mass-screening strategy. WTP was, unsurprisingly, positively associated with the presence of symptoms or other diseases. However, the well-being of the child was not found to be a significant factor explaining WTP.

Other methodologies can be used to assess the burden and the costs of celiac disease. For example, Hershcovici et al. [20] measure the gain in quality-adjusted life-years (QALYs) implied by a screening of the healthy young adult population for celiac disease. Another study using this methodology shows that fecal microbiota transfer for inflammatory bowel disease is cost effective [53], and this treatment was shown to also work in one of the rare cases of refractory celiac disease [6].

## Survey design

An online survey^1^ was conceived to be passed among celiac patients and gather information to measure costs of celiac disease as well as understand the factors influencing those costs. The questionnaire was sent by email to all members of the celiac disease association in the French-speaking part of Switzerland and published on specific social media groups in June 2019. It had previously been tested on a small group and validated by academic experts. The questionnaire is composed of 3 parts: the first part gathers information on how patients deal with their disease and how strongly it affects their daily life. The second part is the CV questions and the third part collects respondents’ socio-economic characteristics.

The contingent question is framed in three steps. The first step is conceived to identify people who would not buy the treatment. It is worded as follows: *“Imagine now that a pharmaceutic lab proposes a new treatment for celiac disease in the form of a daily pill. This pill would prevent the body’s auto-immune reaction following the ingestion of gluten and would thus allow you to eat normally, without danger nor physical issues. The pill would have no side effects. Before answering, think about the advantages and disadvantages of celiac disease and its current treatment, the gluten-free diet. Would you be willing to buy this treatment?”.*

If the answer is “No”, then the respondent is guided to a follow-up question aiming at distinguishing protest bidders from zero bidders. The literature indeed points out the need to treat protest bidders differently from zero bidders [23]. Since their answer is not driven by their WTP, but by other concerns, treating protest bidders as zero bidders would underestimate the mean WTP. In our case, the questionnaire revealed one main protest reason (9 occurrences): the fact that the treatment should be paid by the health insurance, which is mandatory for all Swiss inhabitants. However, respondents also had the choice to openly specify their own reason. This open-ended question allowed to identify 2 respondents who did not believe or trust the contingent scenario. These individuals were treated as protests as well.

The second step of the contingent question aims at eliciting WTP. There are several ways to proceed in the literature, which all have advantages and disadvantages. The chosen elicitation method is a payment card system. Although this method suffers from the anchoring problem, the lower cognitive burden and the statistical efficiency compared to the open-ended and dichotomous-choice format, respectively [1, 2], led to the choice of this elicitation instrument. Another bias linked with payment card is the range bias [50]. Respondents could indeed be influenced by the range of amounts that are suggested to them. However, a study [46] found no range bias in their test with different range of amounts, and rather concluded that answers are affected when the truncation appears too low. To deal with the problem of low truncation, we suggest 7 amounts from CHF 0 to 500 (approx. the same in USD) and leave an open-ended question if the WTP is higher. Respondents willing to pay 0 are guided to the protest bidders identifying question.

We paid particular attention to reducing well-known biases inherent in CV. First, the hypothetical bias may lead to an overstatement of WTP Loomis [31]. Participants might indeed say they would purchase the new treatment even though they would not in reality. To deal with this issue, we follow both an ex ante and ex post procedures. Ex ante, following Kotchen and Reiling [25], we remind respondents about their income constraint. Ex post, we use Blumenschein et al. [8] procedure and insert a third step in our contingent question by asking another follow-up question aiming at measuring the certainty of the answer. This technique was also used by van den Berg et al. [5] in a study about patients’ valuation of their time. According to Ryan et al. [47], calibrating for uncertainty is important since it reduces the difference between actual and hypothetical WTP.

A concern about strategic behavior and incentives has been discussed in the literature (for example: [15]. Indeed, there seems to be a tendency of contributing less to a public good (free-riding), when using an open question, which would be avoided by using a referendum type of question [12]. However, in the present scenario, as we are valuing a private good (exclusion and rivalry are present), this type of bias should not be a problem. Furthermore, the health economics literature usually finds no strategic bias [24].

## Descriptive statistics

A total of 233 people took part in the survey; 170 completed it and 162 corresponded to the criteria of inclusion (biopsy and/or blood test, being over 18, living in Switzerland and having no missing values or major inconsistent answers). 87% of the answers are from members of the celiac disease association.

The representativity of the sample is difficult to assess since characteristics of people suffering from celiac disease in the whole population are not available. In our sample, 75% of the respondents are women and 25% are men, which seems to confirm that the disease is more prevalent among the female population [49]. The average age of respondents is 44 years old but is much lower among non-members of the association (36). This is due to the fact that those who were not members were only recruited via social media.

Respondents are more educated than the Swiss average with 37% of respondents having completed higher education compared to 29% in the population. The professional secondary training (apprenticeship) is underrepresented with only 25% of respondents having completed it, compared to 36.4% of the population OFS [40]. This is not a big surprise since people with higher education are more likely to answer surveys of this type in general.

The median gross household income in the sample is CHF 9000. The distribution seems similar to the Swiss resident population OFS [41]. The average gross household income in the sample is CHF 9006, which is slightly lower than the Swiss average, which was around CHF 10,000 over the past years (2012–2016) OFS [38].

**Fig. 1:**
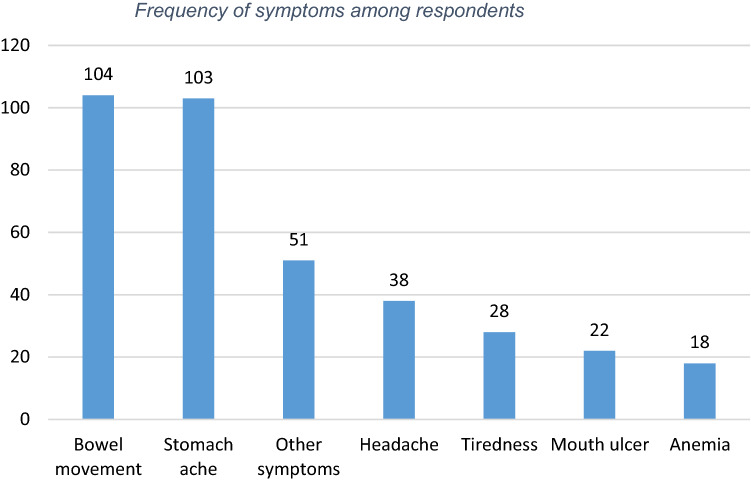
Frequency of symptoms among respondents

Their general health state was judged good or very good by 90% of the respondents. Those who have either other diseases or allergies/intolerances have a lower health on average. 15% of the respondents report none or very mild symptoms before diagnosis. The symptoms range is quite large confirming the observed variety of symptoms in the previous literature. Bowel movement problems and stomachache seem to be the most common as shown in Fig. 1.

The variables used in the empirical analysis are presented in Table 2. The question numbers are indicated in parentheses, when the variable directly results from the questions^2^. Computed variables are explained in the text and in Table 8, in the Appendix.

**Table 2:** Descriptive statistics

Variable	Mean	Std. Dev	Min	Max
Income (Q26a)	4695	3288.95	500	12,001
Extracost (Q15)	102	82.17	0	450
Labor market indirect costs	0.17	0.38	0	1
GFDhealthy (Q12_7)	0.55	0.50	0	1
Logistics (Q12_2)	0.40	1.32	− 2	2
Membership (Q25)	0.88	0.33	0	1
CDAT (Q9)	13.0	3.13	7	24
Symptoms (Q11a*Q11b)	1.44	2.67	0	16
Symptoms before diag. (Q8a)	2.85	1.27	0	4
Diagnosis date (Q7)	2004	11.61	1957	2019
Holiday criterion (Q12_4)	0.40	0.49	0	1
New friends (Q12_6)	0.09	0.29	0	1
Friends/family support (Q12_1)	0.93	0.26	0	1
Cooking talent (Q12_5)	0.55	0.50	0	1
Time spent cooking (Q13)	6.30	3.59	0.5	13

Question number in parentheses

*Income* corresponds to the monthly gross personal income. We decided to include the personal rather than household income in the empirical analysis but results are qualitatively similar when using the household income but less significant. *Extracost* is the amount that respondents pay extra for gluten-free food, per month and per person. These extra costs are considerable compared to the personal income, since 2%, on average, are spent specifically for the purchase of gluten-free food. This amounts to 102 CHF on average, 450 being the maximum in our sample.

*Labor market indirect costs* is a dummy variable indicating whether the respondent faces professional difficulties linked with celiac disease. It takes the value of 1 if the respondent has missed workdays over the last three months due to celiac disease, if the respondent would increase her worktime if she did not suffer from celiac disease, if the diet is a criterion to accept a new job opportunity, or if a treatment would offer more job opportunities. 17% of our sample faces labor market indirect costs.

*GFDhealthy* is a dummy variable, which indicates whether respondents find GFD healthier than regular diet. It is based on a Likert scale, and if the statement “*The gluten-free diet makes you eat more healthily.”* Is answered by either “somewhat agree” or “strongly agree”, it is coded as 1. Overall, 55% of respondents find GFD healthier.

*Logistics* is a categorical variable, based on the same scale as the previously described *GFDhealthy* with the statement “You regularly face logistic difficulties caused by your particular diet.” Logistic difficulties seem to be an issue that a majority of our sample faces. 69% strongly or somewhat agree with the statement.

*Membership* is a dummy indicating whether the respondent is a member of the celiac disease association. 88% of the final sample are members.

**Fig. 2:**
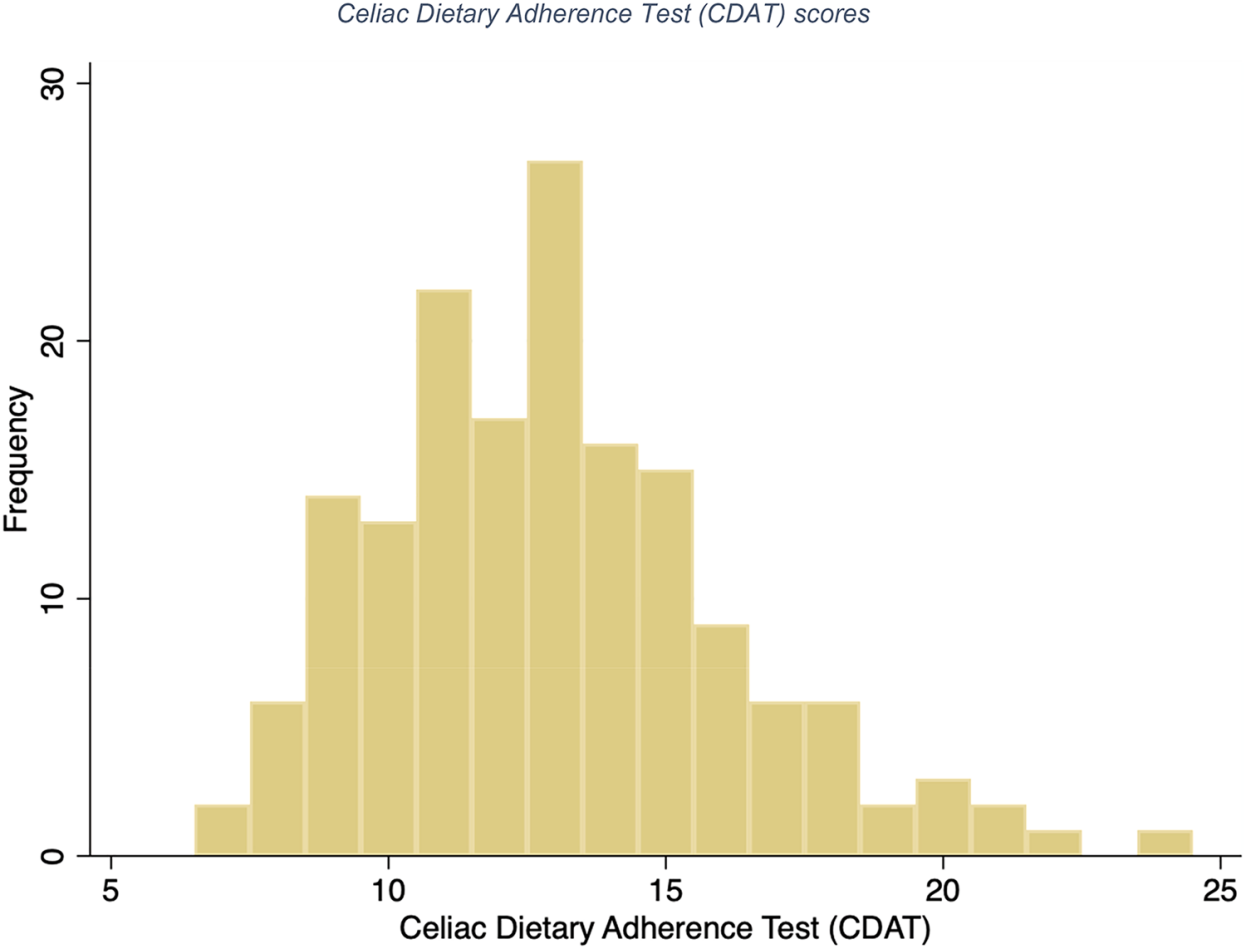
Celiac dietary adherence test (CDAT) scores. Source: questionnaire, see questions Q9a to Q9g. Indicator from Leffler et al. [28] Higher scores indicate lower adherence to the GFD. A score under 13 is associated with excellent or very good adherence. A score over 17 is associated with fair to poor adherence

The celiac Dietary Adherence Test (*CDAT*) measures the adherence to the diet and was computed with the indicator by Leffler et al. [28], which is composed of 7 questions each getting from 1 to 5 points depending on the answer. Therefore, the total score goes from 7 to 35, the higher the score, the poorer the adherence. Figure 2 shows the distribution of the score among respondents. 45% of them had an excellent or very good adherence to the diet (score under 13) and only 5% a fair to poor adherence (a score over 17).

*Symptoms* is an interaction variable between the frequency and the strength of symptoms over the last 4 weeks. 66% had no symptoms while only 3% had symptoms most of the time. Among the respondents facing symptoms, 11% had rather strong symptoms.

*Symptoms before diag* indicates the strength of symptoms before the diagnostic was made. 73% of respondents had strong or very strong symptoms.

*Diagnosis date* indicates the date when the diagnosis was made. It ranges from 1957 to 2019, with an average in 2004.

*Holiday criterion, New friends, Friends/family support* and *Cooking talent* are all dummy variables, based on a Likert scale and coded similarly to the *GFDhealthy* variable. They indicate if the statement “*The availability of gluten-free food is a criterion to choose your holiday destination.“, “The gluten-free diet/celiac disease has allowed you to make new friends.”* or “*The gluten-free diet has developed your cooking talent.*”, respectively, apply to the respondent. 40% choose their holiday destination depending on the availability of gluten-free food, 9% think that celiac disease allowed them to make new friends and 55% think that it improved their cooking talent. Support from family members or friends seems to be very high, as 93% of respondents (somewhat or strongly) agree with the statement that their close friends or family are understanding of their special needs required by the diet.

A third of the respondents had involuntarily eaten gluten at least once during the past four weeks, suggesting that the GFD is still a daily challenge and that the awareness of restaurant staffs or cross-contamination issues could probably be improved. On the other hand, 20% of respondents had eaten gluten voluntarily during the same time span. Unsurprisingly, a negative correlation is observed between the number of accidental/voluntary gluten absorptions and their adverse impact on respondent’s health.

## Empirical analysis

11 protest bidders were identified in our sample, which corresponds to a proportion of 7%. This low proportion is probably due to respondents’ interest in the survey. Since respondents self-select, it is indeed likely that only interested people took the survey. To avoid an extra selection bias potentially arising when dropping protest, we compare protest characteristics with non-protests. Protests are no different on the basis of socio-economic variables. Furthermore, a probit regression revealed no impact of variables related to respondent’s disease on the probability to protest. This check allow us excluding selection bias in this case and safely dropping protest answers from the sample [9].

Regarding the hypothetical bias, there is no consensus on how to deal with uncertainty [32]. To stay conservative, we take advantage of the question asking respondents whether they are sure to be willing to pay the amount chosen, and we provide another estimate of WTP, for which all uncertain respondents’ WTP have been reduced by 50%.

In addition to the mean WTP resulting directly from the answers, we perform multinomial regressions, to identify potential determinants of WTP. Theoretically, as explained in the literature review section, WTP reveals celiac disease patients’ preferences and income. It is also influenced by the physical, social and monetary inconveniences linked with celiac disease as well as by potential ancillary benefits from the GFD. We choose the specifications accordingly and propose different models. We base our favorite model on overall and variable specific significance levels, as well as on the Bayes Information Criteria (BIC), to foster parsimony.

The most parsimonious parametric model, recommended by the BIC is the following:

$$WT{P}_{i}=\alpha + {\beta }_{1}Incom{e}_{i}+{\beta }_{2}Extracos{t}_{i}+{\beta }_{3}Labourcosts+{\beta }_{4}GFDhealth{y}_{i}+{\beta }_{5}Logistic{s}_{i}+{\epsilon }_{i,}$$

where *WTP* is the willingness to pay for the celiac disease treatment, *Income* is the respondent’s gross personal income, *Extracost* is the extra cost stated by the respondent for the GFD, *Labourcosts* is a dummy variable indicating whether the respondent faces indirect costs in her professional activity, *GFDhealthy* indicates whether respondent think the GFD allows her to eat more healthy and *Logistics* indicates whether the respondent faces regular logistical difficulties linked with her diet.

The fact that linear models like Ordinary Least Squares (OLS) predict a dependent variable on both negative and positive values is an important drawback in our case. Indeed, since our valuated good is private, respondents have the choice to purchase it or not and would not need to be compensated. There is, therefore, no reason to state negative WTP. Hence prediction of negative WTP should be ruled out. The importance of the WTP statistical distribution has been highlighted in [10]. A Generalized Linear Model (GLM) using a Gaussian and Gamma distributions with log link, which is able to model, and hence predict, a non-negative continuous variable is an alternative to OLS models. However, given their simplicity, OLS models are preferred to highlight potential determinants of WTP. As robustness checks, we provide coefficients and prediction of WTP using GLM models as well as WTP estimates resulting from the Kaplan–Meier non-parametric estimator [2].

## Results

**Fig. 3:**
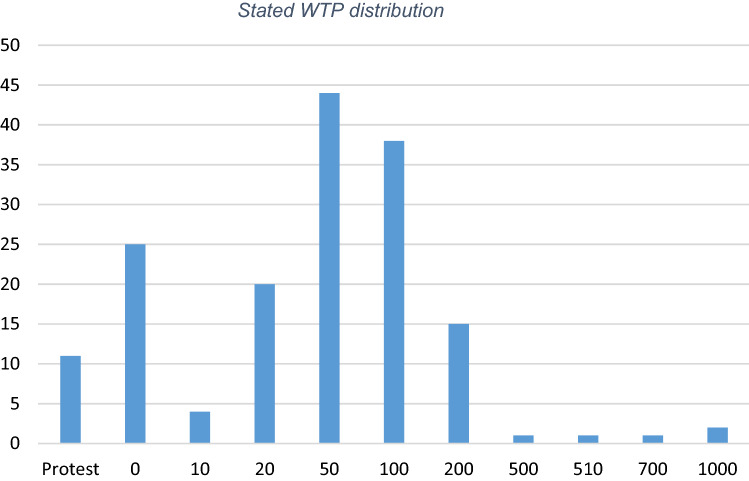
Stated WTP distribution

Among respondents, excluding protest bidders, 83% were willing to pay a positive amount for the treatment and 17% were considered real zeros. The positive bidders were willing to pay CHF 10 up to CHF 1000 per month. As presented in Table 3, mean WTP is CHF 87 and the median CHF 50. Figure 3 shows the distribution of the stated willingness to pay (WTP). As explained in Sect. 5, we also present a corrected WTP (WTP2) taking into account respondents’ uncertainty by dividing uncertain respondents’ WTP by 2. 25% of positive bidders were unsure about their answer.

**Table 3:** WTP distribution resulting directly from the payment card question

	Obs	Median	Mean	Std dev	Min	Max	CI 90%
WTP	151	50	87.09	140.32	0	1000	(68.19; 105.99)
WTP2	151	50	74.75	137.32	0	1000	(56.90; 92.60)

The main advantage of this hypothetical treatment, for more than 80% of the respondents, was “easier organization”, followed by “less frustration” and “be able to eat more palatable food”. An answer that was not suggested came up several times (*n* = 14) in the comments: having an easier social life.

As expected, willingness to pay is positively correlated to personal income: a 1000 CHF increase in annual income would lead to a 7–8 CHF increase in WTP. However, household income and equivalent household income are less significant (results not shown). This is quite surprising as health is generally a shared “cost” among families or households: it was, therefore, expected to be better linked. This is perhaps due to the format of the question. It was asked personally to the respondent, who is not in a position to discuss it with other members of the household whether they would agree to pay for this hypothetical medicine.

Unsurprisingly, the extra costs of the GFD borne by celiac respondents also influence WTP positively. One CHF extra costs would lead to a 0.4–0.5 CHF increase in WTP. The magnitude of this coefficient may seem surprising, since, if respondents integrated all costs in their WTP, all increases in costs should be reflected 1 to 1 in WTP. However, the smaller than 1 coefficient may be explained by unobserved positive effects of the GFD (respectively, negative effects of the pill). For example, it could be explained by the fact that respondents might be reluctant to trade gluten-free food for swallowing a pill every day. The medicine would, therefore, only be a weak substitute to gluten-free food.

Indirect costs borne by respondents on the labor market also have an important impact on WTP. Respondents who answered that they would have better work conditions without celiac disease have a higher WTP ceteris paribus. In comparison with the mean WTP, the difference is considerable being between 37 and 51 CHF per month.

An interesting finding is that those who agree with the statement that GFD is healthier are willing to pay significantly less than the others. Respondents who think that the GFD is healthy are willing to pay 42–59 CHF less per month.

Agreeing with “experiencing regular logistical/organizational difficulties” or “choosing holiday destinations according to the availability of gluten-free food” is significant in some specifications. People agreeing with either statement would be willing to pay around CHF 18 more. Furthermore, the biggest advantage of the medicine is “easier organization”, which confirms the importance of logistical costs in celiac patients’ life.

Table 4 below shows the potential determinants of willingness to pay in different multivariate linear regression models. Coefficients can be interpreted directly as the effect of a linear increases of the independent variables on WTP^3^. Based on the AIC or BIC, our preferred specifications are (1) and (3).

**Table 4:** Determinants of WTP

WTP	(1)	(2)	(3)	(4)
Income (Q26a)	0.0073**	0.0073**	0.0075**	0.0070**
	(0.00)	(0.00)	(0.00)	(0.00)
Extracost (Q15)	0.47***	0.48***	0.41***	0.42***
	(0.13)	(0.13)	(0.13)	(0.13)
Labor market indirect costs	50.97*	50.58*	40.29	37.07
	(27.41)	(27.50)	(26.84)	(27.11)
GFDhealthy (Q12_7)	− 43.00**	− 42.34**	− 58.93***	− 58.81***
	(20.49)	(20.24)	(20.48)	(21.26)
Logistics (Q12_2)	17.51**	18.18**	10.42	11.91
	(7.95)	(7.98)	(8.14)	(8.26)
Membership (Q25)		57.33*	62.74**	62.31*
		(31.76)	(30.92)	(31.57)
CDAT (Q9)		5.99*	6.62**	6.83**
		(3.34)	(3.25)	(3.37)
Symptoms (Q11a*Q11b)		− 2.71		
		(4.02)		
Symptoms before diag. (Q8a)			19.67**	20.35**
			(8.62)	(8.62)
Diagnosis date (Q7)			1.45	1.56*
			(0.93)	(0.93)
Holiday criterion (Q12_4)			44.52**	50.41**
			(21.80)	(22.74)
New friends (Q12_6)			− 62.00*	− 66.26*
			(35.84)	(36.43)
Friends/family support (Q12_1)				31.81
				(39.25)
Cooking talent (Q12_5)				− 0.40
				(21.72)
Time spent cooking (Q13)				− 2.45
				(3.02)
Constant	12.86	− 113.4*	− 3088.3	− 3322.3 *
	(24.75)	(59.07)	(1868.61)	(1875.33)
Observations	151	151	151	151
*R*^2^	0.20	0.24	0.29	0.29
*Adjusted-R*^2^	0.18	0.19	0.23	0.22
*AIC*	1900.0	1899.9	1895.6	1900.3
*BIC*	1921.2	1930.1	1934.9	1948.6

*Standard errors* in parentheses

Variables and their recoding are described in the Appendix

*p < 0.1, **p < 0.05, ***p < 0.01

As expected, willingness to pay is positively correlated to personal income: a 1000 CHF increase in annual income would lead to a 7–8 CHF increase in WTP. However, household income and equivalent household income are less significant (results not shown). This is quite surprising as health is generally a shared “cost” among families or households: it was, therefore, expected to be better linked. This is perhaps due to the format of the question. It was asked personally to the respondent, who is not in a position to discuss it with other members of the household whether they would agree to pay for this hypothetical medicine.

Unsurprisingly, the extra costs of the GFD borne by celiac respondents also influence WTP positively. One CHF extra costs would lead to a 0.4–0.5 CHF increase in WTP. The magnitude of this coefficient may seem surprising, since, if respondents integrated all costs in their WTP, all increases in costs should be reflected 1 to 1 in WTP. However, the smaller than 1 coefficient may be explained by unobserved positive effects of the GFD (respectively, negative effects of the pill). For example, it could be explained by the fact that respondents might be reluctant to trade gluten-free food for swallowing a pill every day. The medicine would, therefore, only be a weak substitute to gluten-free food.

Indirect costs borne by respondents on the labor market also have an important impact on WTP. Respondents who answered that they would have better work conditions without celiac disease have a higher WTP ceteris paribus. In comparison with the mean WTP, the difference is considerable being between 37 and 51 CHF per month.

An interesting finding is that those who agree with the statement that GFD is healthier are willing to pay significantly less than the others. Respondents who think that the GFD is healthy are willing to pay 42–59 CHF less per month.

Agreeing with “experiencing regular logistical/organizational difficulties” or “choosing holiday destinations according to the availability of gluten-free food” is significant in some specifications. People agreeing with either statement would be willing to pay around CHF 18 more. Furthermore, the biggest advantage of the medicine is “easier organization”, which confirms the importance of logistical costs in celiac patient’s life.

Adherence to the GFD, as measured by the indicator CDAT appears to be a significant determinant of the willingness to pay in only one specification: the less adherent, the more willing to pay. This would imply that people who do not follow too strictly their GFD see it as more of a burden than those who strongly comply.

Being a member of the celiac disease association is a significant positive determinant of the willingness to pay. Members are ready to pay, depending on the specification, 57–63 CHF more per month than non-members. Keeping in mind that non-members are underrepresented in the sample, it seems that active involvement with the disease translates into higher motivation to invest in an alternative treatment. The fact that members also pay CHF 65 every year for the membership to the association hints in this direction, although membership also brings other opportunities.

Current symptoms seem not to have an impact on willingness to pay contrary to having symptoms before diagnosis, which is highly significant in all specifications and also of a large magnitude (around 16 CHF for each degree of intensity). This probably indicates that people, who suffered from strong physical symptoms follow the GFD more strictly and thus suffer from less symptoms currently.^4^ However, they remember their symptoms before the diagnostic and might be keener on having a treatment that would not involve unintentional accidents. The self-assessed health state is not a significant determinant, neither is having another disease nor having another food-related allergy or intolerance.

On the one hand, the GFD has ancillary social benefits, such as giving the possibility to make new friends, which significantly decrease WTP from about 62–66 CHF. On the other hand, social costs seem not very important in the determination of WTP, since support from family and friends is not a statistically significant determinant of the willingness to pay. However, this does not necessarily imply that it is not an important factor in the well-being of the patients. Indeed, as there is little variation in this item among respondents it probably does not statistically explain the amount people are willing to pay.

Finally, the fact that the GFD improved cooking talent and the time spent cooking are no significant determinants of WTP. This is not surprising, since acquired cooking talent would not diminish after buying the hypothetical treatment, although it might not further improve. In addition, respondents do not report spending more time on cooking than the regular population according to OFS [38].

Coefficients resulting from the GLM estimations of specification (1) are available in Table 9 in the appendix and predictions of WTP using these GLM coefficients are presented in Table 5. As wished, predicted WTP are bounded between 0 and 1000. The resulting predicted mean WTPs confirm the previous estimates at around CHF 82–84. Interestingly, predicting WTP for protest bidders using the OLS or GLM models results in a slightly higher mean WTP. This difference, however, is not significant, again indicating that protests do not behave differently from non-protests.

**Table 5:** Predictions of WTP using GLM models

		Obs	Median	Mean	Std dev	Min	Max
Gaussian	WTP	151	56.85	81.69	90.61	11.67	969.33
Gamma	WTP	151	72.31	83.58	40.42	13.88	263.84

**Fig. 4:**
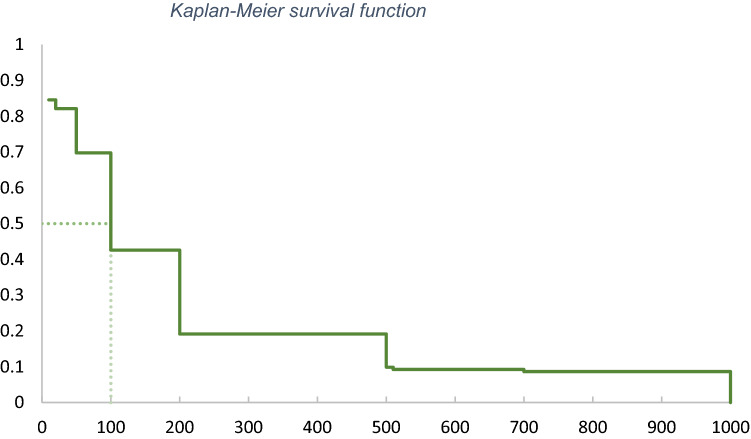
Kaplan–Meier survival function

We provide the results of the Kaplan–Meier estimator, whose survival function is presented in Fig. 4. The Kaplan–Meier mean WTP estimator, presented in Table 6 confirms the magnitude of the WTP estimated above. However, the low number of observation and the resulting wide confidence intervals do not allow to draw many more conclusions from this estimator.

**Table 6:** WTP resulting from the Kaplan–Meier estimator

	Obs	Median	Mean	Std error	CI 90%
WTP	151	100	148.98	92.34	(− 2.92; 300.87)

## Discussion

Without questioning the validity of our results, several limitations have to be acknowledged. Although the general social characteristics are not too different from the general population, we cannot exclude some kind of self-selection bias among respondents since the survey, which hinted at an economic study of the costs of celiac disease and might have attracted less people who do not think it is costly. Some debriefing questions also suggest that respondents did not answer completely rationally or did not fully understand some questions. When they were asked a follow-up question of how much they would pay if the gluten-free diet did not imply an additional cost, the difference between the first and the second answer was not necessarily equal to the amount they had indicated as costs for the diet. Given the complexity of this question, we confidently decided to drop it from the analysis.

Assessing the total costs of a disease is crucial in a cost–benefit framework. For celiac disease, in particular, a measure of costs can provide policy-makers with arguments related to food policy. A strict enforcement of the ordinance on foodstuffs^5^ is indeed key to make sure celiac patients are not contaminated with gluten when they eat outside of home. This ordinance makes it compulsory for restaurant to provide accurate information on the presence of allergens—including gluten—in the meals they prepare. This could also be accompanied with a campaign raising awareness about what is gluten, where it can be hidden in processed food, and how to prepare a meal avoiding cross-contamination. The current annotation of the allergens in bold on packages is already very helpful for celiac patients when shopping, but in restaurants, the list of ingredients is generally not disclosed on the menu. The staff should, therefore, be sufficiently educated on this topic to be able to give the correct information to patients, just like cooks should have, in their training, a module on cooking with different allergens.

These measures would reduce the frequency of contamination of celiac patients, thereby increasing their adherence and lowering complications. This would also reduce intangible costs, as it would be less stressful to eat outside of home for celiac patients.

The food industry could also consider these results to shape its pricing policies or supply a bigger variety of gluten-free products. If respondents are willing to pay for a treatment, it is likely that they are also willing to pay for more palatable and more varied gluten-free food. A better availability of gluten-free food would allow a much easier organization and would probably increase adherence to GFD, which in the end, would reduce intangible costs.

The pharmaceutical industry could use this WTP information to decide whether to invest in research and development. Indeed, the most effective way of reducing the costs in the long run would probably be to find a cure to this disease. Therefore, ongoing efforts towards a medication should continue and the funding of the research is necessary.

Other contingent valuations on the burden of celiac disease would allow for interesting comparisons over time or across countries. In addition, another contingent valuation could be organized with another elicitation method to have a second estimate of the private costs in Switzerland.

## Conclusion

This paper proposes a first monetary measure of the private costs, including intangible costs, of celiac disease in Switzerland thanks to a contingent valuation. Respondents were willing to pay CHF 10 up to CHF 1000 per month for the treatment. Mean WTP is found to be CHF 87 (75–150) per month or CHF 1044 per year. This amount compares with costs of other illnesses. Celiac disease patients seem to be willing to pay less than prostate cancer patients in Li et al. [29] or multiple sclerosis patients [30] but a similar amount to atopic dermatitis [4]. These results are unsurprising and confirm the fact that celiac disease is costly but its less disabling symptoms or the existing GFD to deal with them explain the relatively low WTP compared to other diseases.

WTP is positively influenced by direct and indirect costs of the disease. In particular, individuals, who spend more on gluten-free food and those facing more constraints on the labor market are willing to pay more than others. The intensity of logistical constraints also affects WTP positively. On the other hand, individuals, who find the GFD healthier are willing to pay less. Finally, unlike symptoms before diagnostic, the current presence or intensity of physical symptoms are found to be insignificant. The latter result can be explained by the fact that, individuals facing stronger symptoms are more likely to adhere strictly to the GFD and, hence, to reduce their frequency.

The results confirm that there are substantial intangible costs that seem to be mainly composed of an impaired social life, a more stressful organization and indirect costs on the labor market, whereas current physical symptoms do not influence WTP.

## Data Availability

Upon request.

## Appendix

### I. Questionnaire

Hello,

Thank you for your interest in this economic study on celiac disease.

By answering the following questions, you accept that the data will be confidentially handled in an academic context and certify that you are 18 years old or older.

The questionnaire lasts approximately 15 min, you can interrupt it at any time. The data gathered until this point could, however, still be taken into account in the analysis.

For any questions, send an email at (email address).

Table 7 lists the survey's question, Table 8 explains how variables are computed, and Table 9 extra estimation results from GLM.

**Table 7:** List of questions and resulting variables

Q	Question	Values	Value labels
Q1	Have you been diagnosed with celiac disease?	0, 1	Yes (1) No (0) END
Q2a	Has the diagnostic been confirmed by a positive blood test?	0, 1	Yes (1) END No (0)
Q2b	Has the diagnostic been confirmed by a biopsy?	0, 1	Yes (1) No (0)
Q3	Have you been diagnosed with other chronic diseases?	0, 1	Yes (1) No (0)
Q3_TEXT	If yes, please precise:	Text	
Q4	Have you been diagnosed with other diseases?	0, 1	Yes (1) No (0)
Q4_TEXT	If yes, please precise:	Text	
Q5	Have you been diagnosed with other allergies or food intolerances?	0, 1	Yes (1) No (0)
Q5_TEXT	If yes, please precise:	Text	
Q6	How would you assess your current health condition?	1 – 5	Very good (1) Good (2) Average (3) Poor (4) Very poor (5)
Q7	When have you been diagnosed with celiac disease? Please indicate the year	yyyy	
Q8a	Before the diagnostic, did you have…	0–4	No symptoms (0) Very light symptoms (1) Light symptoms (2) Strong symptoms (3) Very strong symptoms (4)
	*Please indicate the type of symptom:*		
Q8b_1	Stomach ache	0, 1	Yes (1) No (0)
Q8b_2	Bowel movement problems	0, 1	Yes (1) No (0)
Q8b_3	Headaches	0, 1	Yes (1) No (0)
Q8b_4	Mouth ulcer	0, 1	Yes (1) No (0)
Q8b_5	Other symptoms	0, 1	Yes (1) No (0)
Q8b_5_TEXT	If other symptoms, please precise:	text	
Q9ab_1	*Over the last 4 weeks*, have you experienced low levels of energy?	1–5	None of the time (1) A little of the time (2) Some of the time (3) Most of the time (4) All of the time (5)
Q9ab_2	*Over the last 4 weeks*, have you experienced headaches?	1–5	“
	*Please indicate if you agree with the following statements:*		
Q9cde_1	I manage to follow a gluten-free diet when I eat outside (not home)	1–5	Strongly agree (1) Somewhat agree (2) Neither agree nor disagree (3) Somewhat disagree (4) Strongly disagree (5)
Q9cde_2	Before doing something I carefully consider consequences	1–5	“
Q9cde_3	I do not consider myself a « failure»	1–5	“
Q9f	How important are accidental gluten ingestions for your health?	1–5	Very important (1) Somewhat important (2) Neutral/Unsure (3) A little important (4) Not at all important (5)
Q9g	Over the last 4 weeks, how many times have you eaten gluten deliberately?	1–5	0 (never) (1) 1–2 (2) 3–5 (3) 6–10 (4) > 10 (5) Don't know (99)
Q10a	Over the last 4 weeks, how many times have eaten gluten accidentally?	1–5, 99	0 (never) (1) 1–2 (2) 3–5 (3) 6–10 (4) > 10 (5) Don't know (99)
Q10b	Is following a gluten-free diet a concern for you?	0–3	Yes, A lot (3) Yes, a little (2) No, not much (1) No, not at all (0)
Q11a	Over the last 4 weeks, have you suffered from physical symptoms caused by the ingestion of gluten?	0–4	None of the time (0) A little of the time (1) Some of the time (2) Most of the time (3) All of the time (4)
Q11b	How strong have these symptoms been?	0–5	None (0) Mild (1) Rather mild (2) Average (3) Rather strong (4) Strong (impeding another activity) (5)
	*Please indicate if the following statements apply to you:*		
Q12_1	Your social circle (family and friends) is sympathetic relative to your particular food requirements	1–5	Strongly agree (1) Somewhat agree (2) Neither agree nor disagree (3) Somewhat disagree (4) Strongly disagree (5)
Q12_2	You regularly face logistic difficulties caused by your particular diet	1–5	“
Q12_3	Your diet is a criterion to accept a new job opportunity	1–5	“
Q12_4	The availability of gluten-free food is a criterion to choose your holiday destination	1–5	“
Q12_5	The gluten-free diet has developed your cooking talents	1–5	“
Q12_6	The gluten-free diet/celiac disease has allowed you to make new friends	1–5	“
Q12_7	The gluten-free diet makes you eat healthier	1–5	“
Q13	How much time on average do you spend cooking breakfast, lunch and dinner, preparing preserves or baking per week?		0.5–13 +
Q14	Do other members of your household usually eat gluten-free at home as well?	0, 1	Yes (1) No (0)
Q15	How much extra does the gluten-free diet costs, compared to a classic diet, per month and per person? *In other words, how much could you spare per month and per person if you had a classic diet?*	9999, 0–110, 8888	gluten-free less expensive, 0, 10, 20, 30, 40, 50, 60, 70, 80, 90, 100, 110 CHF 110 + CHF
Q15b	[If more than 110 CHF] Indicate the amount:	> 110	
Q16a	Imagine now that a pharmaceutic lab proposes a new treatment for celiac disease under the form of a daily pill. This pill would prevent the body’s auto-immune reaction following the ingestion of gluten and would thus allow you to eat normally, without danger nor physical issues. The pill would have no side effect Before answering, think about the advantages and disadvantages of celiac disease and the diet, which you follow. Would you be willing to buy this treatment?	0, 1	Yes (1) No (0)Why?
Q16b	To answer this question, please be aware that you will be responsible to pay the treatment, not your insurance company. Think about the advantages of the treatment but keep in mind that your budget is limited. How much would you be willing to pay per month for this treatment?	0, 10, 20, 50, 100, 200, 500, 8888	0 CHFWhy? 10 CHF, 20 CHF, 50 CHF, 100 CHF 200 CHF, 500 CHF, MoreHow much?
Q16b2	You are willing to pay more than CHF 500 per month. Please specify the amount that you would be willing to pay	> 500	CHF
Q16c	I am sure to be willing to pay this amount if the treatment existed	0, 1	Yes I’m sure (1) No, I’m not sure (0)
	Why? If the respondent is not willing to buy or is willing to buy and pay 0 CHF		
Q16d_1	My insurance should pay for it	0, 1	
Q16d_2	I don’t like treatments with medicines	0, 1	
Q16d_3	The current treatment (gluten-free diet) suits me	0, 1	
Q16d_11	I don’t have the financial means	0, 1	
Q16d_12	Other	0, 1	
Q16d_12_TEXT			
	*What advantages would this treatment bring you?*		
Q17_1	Less stress	0, 1	
Q17_8	Easier organization	0, 1	
Q17_2	More free time	0, 1	
Q17_3	More job opportunities	0, 1	
Q17_4	Be able to eat more palatable food	0, 1	
Q17_5	Be able to eat more healthily	0, 1	
Q17_6	Less frustration	0, 1	
Q17_7	Other advantages	0, 1	
Q17_7_TEXT		Text	
Q18	You are willing to pay ${16b/ChoiceGroup/SelectedChoicesTextEntry} per month for this treatment. If the gluten-free diet did not imply extra costs compared to the classic diet, would your willingness to pay diminish? In other words, if, by taking this new treatment, you could not spare money on food by eating normally, would you be willing to pay less for the treatment?	0, 1	Yes (1) WTP2 No (0)
Q18a	In that case, how much would you be willing to pay?	#	
Q18b	I am sure to be willing to pay this amount for the treatment if the gluten-free diet implied no extra costs	0, 1	Yes I’m sure (1) No, I’m not sure (0)
Q20a	Over the last 3 month, have you missed workdays because of celiac disease symptoms? Please specify how many missed days	0–30, 35.5, 45.5, 55.5, 65,5, 75.5, 85.5	0, 1, 2, …, 29, 30, 31–40, 41–50, 51–60, 61–70, 71–80, 81–90
Q20b	If you did not have celiac disease, would you increase your worktime? Please specify how much more time you would work	0–100	0%–no increase 10%–half a day 20 20%–1 day 30%–1.5 days 40%–2 days 50%–2.5 days 60%–3 days 70%–3.5 days 80%–4 days 90%–4.5 days 100%–5 days
	*Please indicate how many times you have needed the following medical services linked with celiac disease over this year*		
Q21a_1	Intestinal biopsy	#	
Q21a_10	Visit by a gastroenterologist for a biopsy	#	
Q21a_3	Visit by a gastroenterologist for other reasons	#	
Q21a_2	Bone densitometry	#	
Q21a_11	Visit by a dietician	#	
Q21a_4	Visit by a nutritionist	#	
Q21a_5	Visit by a general practitioner	#	
Q21a_6	Iron injection	#	
Q21a2	Other medical service, please precise	Text	
	*Over the last 4 weeks have you taken medicine to lighten symptoms of celiac disease? Please specify how many times you have taken them.,*		
Q21b_4	Painkiller	#	
Q21b_9	Gastritis medication	#	
Q21b_5	Anti-inflammatory	#	
Q21b_6	Iron supplements	#	
Q21b_7	Calcium supplements	#	
Q21b_8	Other	#	
Q21c_1	I have not needed any medical service nor medication against celiac disease over this year	0, 1	(tick box)
Q23a	Your age	> 18	
Q23b	Gender	0, 1	Men (0) Women (1)
Q23c	Your level of education:	1–5	Compulsory school (1) Professional secondary school (2) General secondary school (3) Higher professional training (4) Higher education (5)
Q23c_10_TEXT	Other education	Text	
Q23d	Main professional activity	Text	
Q23e	What is your professional activity rate? If you do not work, please answer 0	0–100, 110	0%, 10%, 20%, 30%, 40%, 50%, 60%, 70%, 80%, 90%, 100%, more
	*Number of people in your household, you included:*		
Q23f_1_TEXT	Adults	#	
Q23f_2_TEXT	Kids (less than 14 years old)	#	
	*What is your professional status?*		
Q23g_1	Employee	0, 1	
Q23g_2	Independent worker	0, 1	
Q23g_3	Unemployed	0, 1	
Q23g_4	Housewife/househusband	0, 1	
Q23g_5	Student	0, 1	
Q23g_6	Other status	0, 1	
Q23g_6_TEXT		Text	
Q23h	How many people in your household have been diagnosed with celiac disease (including you)?	0–10	
Q24	Canton of residence	19–45	[List of Swiss cantons] 22 Bern 25 Fribourg 26 Genève 29 Jura 31 Neuchâtel 39 Ticino 41 Vaud 42 Valais 45 Autre pays
Q25	Are you a member of the Romandie celiac disease association?	0, 1	Yes (0) No (1)
Q26a	How much is your monthly gross personal income, on average? *NB: your gross income corresponds to your income before social deductions and before taxes*	500, 1500, 2500, 3500, 4500, 5500, 6500, 7750, 9250, 11,000, 12,001	1000 CHF or less per month 1001–2000 CHF per month 2001–3000 CHF per month 3001–4000 CHF per month 4001–5000 CHF per month 5001–6000 CHF per month 6001–7000 CHF per month 7001–8500 CHF per month 8501–10,000 CHF per month 10,001–12,000 CHF per month More than 12,000 CHF per month
Q26b	How much is the monthly gross income of your household, on average? *NB: your gross income corresponds to your income before social deductions and before taxes*	1000, 3000, 5000, 7000, 9000, 11,000, 13,000, 15,500, 18,500, 20,001	2000 CHF or less per month 2001–4000 CHF per month 4001–6000 CHF per month 6001–8000 CHF per month 8001–10,000 CHF per month 10,001–12,000 CHF per month 12,0001–14,000 CHF per month 14,001–17,000 CHF per month 17,001–20,000 CHF per month more than 20,000 CHF per month

Thank you for your participation, which will be very useful to assess the quality of life of persons with celiac disease and the costs associated to this disease

Would you be willing to be contacted again to receive the results? YES/NO, email address: …

Comment: …

For any question or comment, you can contact me at the following address: (email address)

**Table 8:** Computed variables

Celiac Dietary Adherence Test	7–35		Indicator for the gluten-free diet adherence (see Leffler et al. [28]). Sum of questions Q9a-g
Additional cost of the gluten-free diet	#	CHF	From Q15 and Q15b
Dummy variable for labor market indirect costs	0, 1		Indirect = 1 if Q20a > 1 or Q20b > 0 or Q12_3 = 1 or Q12_3 = 2 or Q17_3 =1
Indicator for frequency and strength of symptoms	0–20		Q11a*Q11b

**Table 9:** Results from the GLM estimation using a Gaussian family and a log link

WTP	Gaussian	Gamma
Income	0.000095***	0.000045
	(0.000025)	(0.000028)
Extracost	0.0024***	0.0042***
	(0.00079)	(0.0012)
Labor market indirect costs	0.66***	0.30
	(0.18)	(0.24)
GFDhealthy (Q12_7)	− 0.65***	− 0.32*
	(0.25)	(0.18)
Logistics (Q12_12)	0.27***	0.15**
	(0.11)	(0.068)
Constant	3.58***	3.73***
	(0.23)	(0.23)
Observations	151	151
*AIC*	1862.7	1615.1
*BIC*	1880.8	1633.2

Standard errors in parentheses

**p* < 0.1, ***p* < 0.05, ****p* < 0.01

## Footnotes

1 The full questionnaire is available in Table 7 in the Appendix.

2 Questionnaire available in the Appendix.

3 Using the uncertainty corrected WTP leads to similar coefficients, both in terms of sign and magnitude.

4 This hypothesis is confirmed by the sign of the correlation between the variable CDAT and the strength of symptoms before diagnostic.

5 Ordonnance du DFI concernant l’information sur les denrées alimentaires (OIDAI), https://www.admin.ch/opc/fr/classified-compilation/20143397/201705010000/817.022.16.pdf.

## Code availability

Upon request.
